# Itinerant Dentist

**Published:** 1845-03

**Authors:** 


*Itinerant Dentist.*—It is stated in a Baltimore daily newspaper, that a man, calling himself a dentist, recently visited Ellicott's Mills, and was employed by many of the citizens of that place to dress up their teeth, and that it was discovered, after his departure, that his operations had not only been productive of "great pain and suffering, but, it was feared, lasting injury." It would be well for suffering humanity, if this was the only place that had suffered from ill-advised and awkwardly performed dental operations. There is hardly a town, village, or neighborhood in the United States, which has not been similarly imposed upon.—*Balt. Ed.*

